# Demographic and clinical characteristics of children seeking psychiatric services in the Nile Delta region: an observational retrospective study

**DOI:** 10.1186/s13033-019-0323-6

**Published:** 2019-10-23

**Authors:** Mohammad A. Seleem, Reham A. Amer, Amr H. Romeh, Hesham M. Hamoda

**Affiliations:** 1 Department of Psychiatry and Neurology, Faculty of Medicine, Tanta University, 31527 Tanta, Egypt; 2 Department of Mental Health, Cwm Taf University Health Board, Wales, UK; 3 Department of Psychiatry, Boston Children’s Hospital and Harvard Medical School, Boston, USA

**Keywords:** Clinical, Sample, Child, Psychiatry, Egypt, Nile, Delta

## Abstract

**Background:**

Epidemiological studies, describing both community and clinical samples of youth in need for psychiatric help, are rare in the middle east. To our knowledge, this is the first study that aims to investigate the demographic and clinical characteristics of a sample of children suffering from emotional and behavioral problems seeking psychiatric services in the Nile Delta region and the largest clinical sample to date in Egypt.

**Methods:**

The files of all new cases who presented for care in the outpatient service for children and adolescents between August 2016 and July 2018 were reviewed. Ninety-six files were excluded due to missing data while another 18 files were found to be for adults (ages > 18 years old), so the sample included 886 cases.

**Results:**

The ages of our sample (n = 886) ranged from 18 months to 18 years with an average of 7.5 (± 3.8) years. Most of our cases were male, school aged children, living within low-income households and predominantly coming from rural areas. The most common diagnoses were attention deficit hyperactivity disorder (ADHD) (22.6%), intellectual disability (ID) (13.7%), depressive disorders (13.3%), and disruptive behavior disorders (DBD) (12.3%). Strong protective effects were found for family integrity and stability. Corporal punishment and physical and sexual abuse were noted to be significant risk factors for internalizing and externalizing disorders in children and adolescents.

**Conclusions:**

Except for males being a majority in our sample of children seeking psychiatric consultation, demographic patterns and prevalence of psychiatric disorders are comparable to other tertiary clinical samples in other parts of the world.

## Background

Various emotional, cognitive, and behavioral disorders are prevalent among young generations all over the world [1]. At any given point of time, it is estimated that the percentage of children suffering from at least one psychiatric disorder ranges between 14 and 20% [2]. The percentage of adolescents receiving mental health services in the US was estimated to be 21.3% [3]. Around half of adult psychiatric disorders might have their onset before the age of 14 [4]. Youth with emotional and behavioral problems are at greatly increased risk of conduct problems, substance abuse, in addition to aggressive and criminal behavior [5]. Mental health problems in youth put a great burden, not only on mental health system, but also on the education, child welfare, and juvenile justice systems [6].

Accurate evaluation of the prevalence rates of psychiatric disorders for youth in both community and clinical settings is fundamental for planning adequate mental health services, developing prevention programs and early detection of characterized psychiatric illness for this vulnerable patient group [7, 8]. Several factors may contribute to conflicting data regarding the exact prevalence rate for this particular group. One of the major determinants of prevalence rate is the selection of the tools, as well as on the nosology and classification. Using reliable, valid, and clinically useful methods for both assessment and diagnosis contribute to more accurate estimations [9]. Additionally, family structure and socio-economic status (SES), and the nature of the informants (teachers, parents, or the child) may also affect the prevalence rates [8, 10].

Common epidemiological studies of childhood mental illness were performed in western populations and the vast majority of these studies have been performed in the United States and the United Kingdom [11]. In comparison, less studies have been done in South America, Europe, Asia, and Africa [7, 9, 12, 13]. In the Arab world, there is a limited number of studies from the Kingdom of Saudi Arabia [14, 15], the United Arab Emirates [16–18], and Egypt [19, 20]. Clinical samples are expected to be different from community samples as only 27% of children with a psychiatric disorder were reported to receive specialized health care [21].

In early 2018, Egypt population was estimated to exceed 96 million people, with approximately 43% of this number aged less than 19 years [22]. In the last few years, child and adolescent mental healthcare is a subject of more attention in Egypt and the Arab world. One of the few community surveys performed in Upper Egypt on children 6–12 years old reported a prevalence rate of 8.5% for any psychiatric diagnosis, 2.0% for emotional disorders; 6.6% for Conduct disorder; and 0.7% for hyperactivity disorder [19]. A smaller clinical sample recruited from a child psychiatry outpatient clinic in Cairo showed that the most commonly diagnosed disorder was ADHD, followed by mental retardation, autism, conduct disorder and finally depression. Most children were referred by relatives, followed by pediatricians and they presented for psychiatric care on average more than 3 years after the onset of their illness [23].

The child and adolescent psychiatry outpatient service in Tanta University was established in 2012, followed by the inpatient unit in 2013, to be the first specialized outpatient and inpatient units serving the wide catchment area in the middle of the Nile delta. This area includes three governorates (Kafr El-Sheikh, Gharbia, and Monofia) with a population of about 13 million people, the majority of which (approximately 75%) lives in rural areas [22]. To our knowledge, this is the first study to investigate the demographic and clinical characteristics of a clinical sample of children suffering from emotional and behavioral problems in the Nile Delta region. This work aims to explore the demographic and clinical characteristics of children and adolescents who present for mental health psychiatric services in this region.

## Methods

This is an observational retrospective study investigating the demographic and clinical profile of a sample of children and adolescents referred for psychiatric consultation in one of the few tertiary centers for child and adolescent psychiatry in the Nile Delta. The sample included all patients aged between 18 months and 18 years old who visited the child and adolescent psychiatry unit in Tanta Psychiatry and Neurology Center during a period of about 28 months between August 2016 and July 2018. All children who were less than 6 years of age at the time of the primary evaluation were considered preschoolers. Those who are 6 years or older but less than 12 years old were considered school-aged children while those who are 12 year or older were considered adolescents.

Data on demographic and socio-economic variables were collected for each family by trained social workers. This included age, gender, birth order, residence area, and child education level. It also included parental marital status, parental education and employment status, family income, and number of children in the household. A detailed developmental, medical, and family history was also obtained by child psychiatry fellows including birth complications, developmental milestones, history of physical or sexual abuse, history of medical illness, need for admission, and family history of psychiatric illness. For technical use, and to differentiate it from corporal punishment, which is common and relatively accepted in most of the Arab communities [24, 25], physical abuse was defined as any corporal punishment that left a mark on the skin for a period of more than 24 h. As both Egyptian law [26] and social norms prohibits any kind of sexual activity with a minor (a person who is less than 18 years old), we considered involving the child in any form of sexual activity, including showing him/her pornographic materials, as a form of sexual abuse.

The second stage was to verify the presence of a psychiatric disorder among the children and adolescents and it included structured psychiatric interview, the MINI International Neuropsychiatric Interview for Children and Adolescents for parents and with children and adolescents except for preschoolers [27] The MINI International Neuropsychiatric Interview for Children and Adolescents (MINI-Kid) is a structured psychiatric interview that administrated in approximately 15–20 min. The MINI has been validated against other structured interviews, and therefore, we used the Arabic version validated on an Egyptian sample [28, 29]. All interviews were administered by trained bachelor’s degree interviewers and were reviewed, and the diagnoses were confirmed by a trained child and adolescent psychiatrist (the first author). Some modifications to the questions of the MINI-kid were done to be consistent with DSM-5 diagnostic Criteria [30]. Psychometric evaluations, mainly the Arabic translation of the Stanford-Binet Intelligence quotient (IQ) fourth edition [31, 32], were used to assess intellectual functions for all cases.

The medical records of one thousand patients presenting to the unit were reviewed. Ninety-six files were excluded due to missing data while another 18 files were found to be for adult cases (age > 18 years old). Those files were excluded and so we reviewed the files of 886 cases.

## Results

In our sample, the age of the children presented to the outpatient unit ranged from 18 months to 18 years, with a mean age of 7.5 (± 3.8) years. About 36.3% of our families were referred by local psychologists, speech therapists, and nurses, 22.6% were referred by schools, 21.1% were referred by primary care pediatricians, and 20% of families were self-referred. Most of cases (48.3%) were school age children (6–12 years old), 34.9% were preschoolers (age less than 6 years), and 16.8% of them were adolescents. Only 31.5% of patients were females and slightly more than half of them (56.6%) came from rural areas. The clear majority of children (90%) were living with both married parents and approximately 75% recorded having a monthly household income of less than 5000 Egyptian pounds (approximately 300 USD). About 10% of the fathers and 11% of the mothers were illiterate while 75.6% of the fathers and 71.7% of the mothers held a college degree. About 6% of the school age children and 7.4% of the adolescents were out of school. Physical punishment was reported in about one-fifth of cases with no significant differences between age categories or genders. Adolescents suffered from the highest rates of sexual abuse followed by younger age groups in a descending order of age. The highest rates of physical abuse were recorded in school age group followed by adolescents and preschoolers. The most likely age category to need admission was adolescents followed by school age children and finally preschoolers (Tables 1, 2). Male and female children showed no significant differences regarding the above-mentioned demographic and basic clinical variables (Additional file 1: Tables S1 and Additional file 2: Table S2).

**Table 1:** Demographic characteristics of children seeking psychiatric medical advice according to age group (n = 886)

Variable	Preschoolers (n = 309, 34.8%)	School age (n = 428, 48.3%)	Adolescents (n = 149, 16.8%)	Total (n = 886)	Statistic	p value
Gender (Female)	92 (29.8%)	131 (30.6%)	56 (37.6%)	279 (31.5%)	x^2^ = 3.1	0.3
Residence						
Rural	179 (57.9%)	251 (58.8%)	72 (48.3%)	502 (56.7%)	x^2^ = 5.2	0.07
Urban	130 (42.1%)	176 (41.2%)	77 (51.7%)	383 (43.3%)		
Family status (living with both parents)	285 (92.2%)	383 (89.5%)	129 (86.6%)	797 (90%)	x^2^ = 3.7	0.2
Father education						
Illiterate	27 (8.7%)	44 (10.3%)	19 (12.8%)	90 (10.2%)	x^2^ = 4.2	0.4
< High school	37 (12%)	66 (15.4%)	23 (15.4%)	126 (14.2%)		
≥ High school	245 (79.3%)	318 (74.3%)	107 (71.8%)	670 (75.6%)		
Mother education						
Illiterate	28 (9.1%)	49 (11.4%)	22 (14.8%)	99 (11.2%)	x^2^ = 4.3	0.4
< High school	58 (18.8%)	73 (17.1%)	21 (14.1%)	152 (17.2%)		
≥ High school	223 (72.2%)	306 (71.5%)	106 (71.1%)	635 (71.7%)		
Family income						
< 5000 EGP/M	238 (77%)	327 (76.4%)	101 (67.8%)	666 (75.2%)	x^2^ = 5.3	0.07
≥ 5000 EGP/M	153 (25.5%)	67 (24%)	48 (32.2%)	220 (24.8%)		
Child education						
Out of school	–	25 (5.9%)	11 (7.4%)	36 (6.3%)	FET	0.8
Regular school	–	391 (92%)	135 (90.6%)	526 (91.6%)		
Special school	–	9 (2.1%)	3 (2%)	12 (2.1%)

**Table 2:** General clinical characteristics of children seeking psychiatric medical advice according to age groups (n = 886)

Variable	Preschoolers (n = 309, 34.8%)	School age (n = 428, 48.3%)	Adolescents (n = 149, 16.8%)	Total (n = 886)	Statistic	p value
Family history of psychiatric illness	41 (13.5%)	75 (17.7%)	21 (14.1%)	137 (15.6%)	x^2^ = 2.7	0.3
History of birth complications	78 (25.7%)^a^	67 (15.9%)^b^	13 (8.7%)^c^	158 (18.1%)	x^2^ = 21.9	*≤ 0.001*
History of delayed milestones	171 (56.4%)^a^	186 (44.1%)^b^	37 (24.8%)^c^	394 (45.1%)	x^2^ = 40.6	*≤ 0.001*
Birth order (5th or more)	4 (1.3%)	10 (2.3%)	3 (2%)	17 (1.9%)	FET	0.6
History of abuse						
Physical punishment	51 (16.8%)^a^	91 (21.6%)^a^	31 (20.8%)^a^	137 (19.8%)	FET	*≤ 0.001*
Physical abuse	8 (2.6%)^a^	35 (8.3%)^b^	7 (4.7%)^a, b^	50 (5.7%)		
Sexual abuse	0 (0.0%)^a^	5 (1.2%)^a, b^	4 (2.7%)^b^	9 (1%)		
Need for admission	5 (1.6%)^a^	34 (7.9%)^b^	31 (20.8%)^c^	70 (7.9%)	x^2^ = 50.9	*≤ 0.001*

Differing superscripts denote significant between-group differences calculated using pair-wise Chi square tests (p-values ≤ 0.05)

**Fig. 1:**
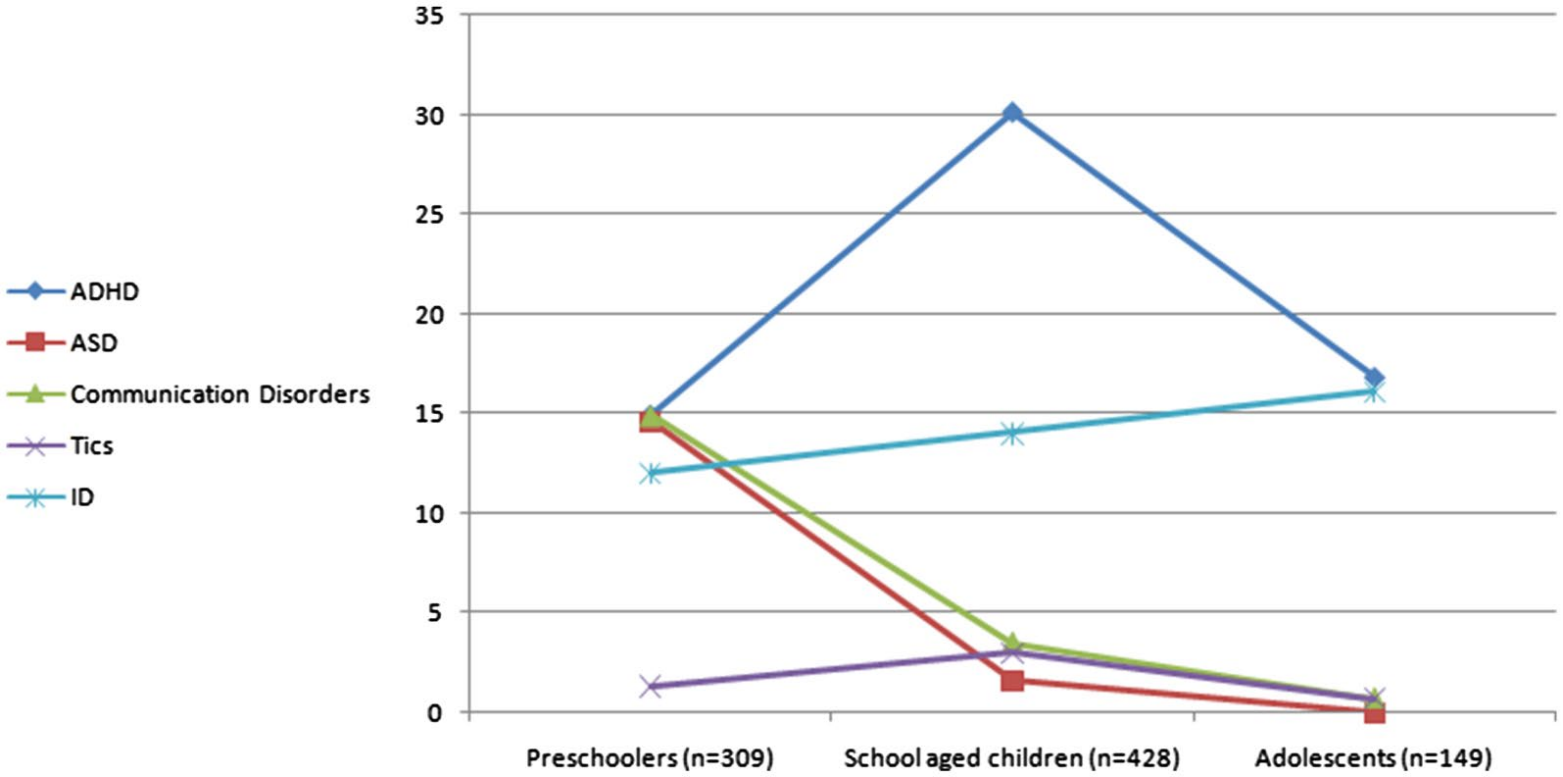
Percentage of children presenting by neurodevelopmental disorders across age groups

**Fig. 2:**
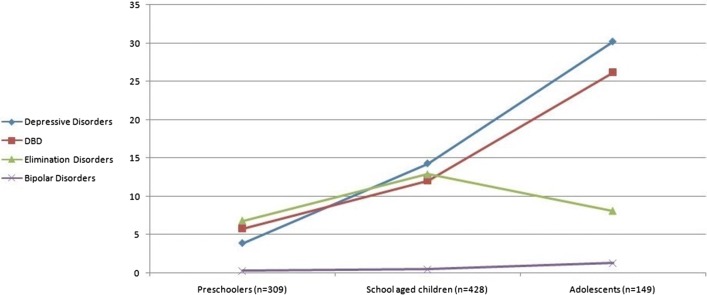
Percentage of children presenting by other relatively common disorders across age groups

**Fig. 3:**
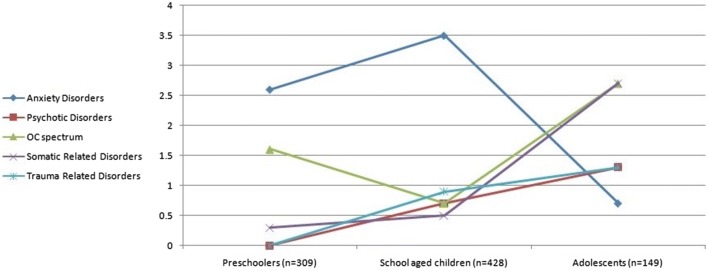
Percentage of children presenting by other relatively rare disorders across age groups

**Fig. 4:**
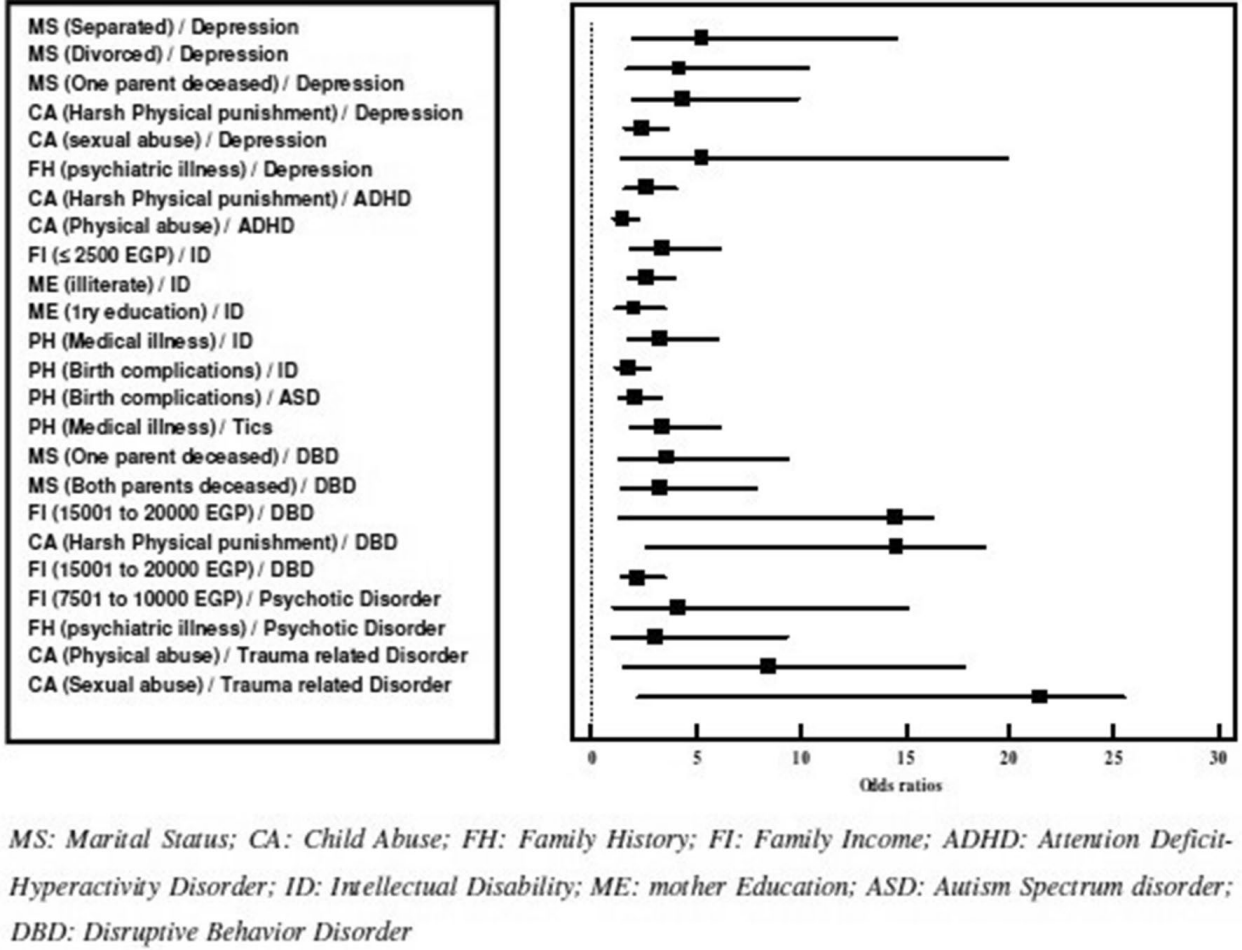
Forest plot showing the significant odds ratios for the associations between some selected demographic/clinical factors and diagnostic categories

Almost one-third (28.7%) of the sample had borderline intellectual abilities (IQ between 70 and 90) while 13.7% were intellectually disabled. The most common diagnoses were attention deficit hyperactivity disorder (ADHD) (22.6%), Intellectual disability (ID) (13.7%), depressive disorders (13.3%), disruptive behavior disorders (DBD) (12.3%), and elimination disorders (9.9%). Only 5.9% of children were diagnosed as having autism spectrum disorder. The percentage of children who received a primary diagnosis of a depressive disorder, disruptive behavior disorder, somatic or trauma-related disorder increased with age i.e. the older the child the more likely the children receive one of these diagnoses. Other diagnoses, such as ADHD, anxiety disorders, and elimination disorders showed a peak in school age children and were less likely to present in preschoolers or adolescents. The rate of seeking help in families with a child on autism spectrum and other communication disorders seems to be falling while the child grows older (Table 3, Figs. 1, 2, 3, 4). A small percentage (9.4%) of children who came to the clinic was suffering from neurological complaints (e.g. migraines and epilepsy) (Table 3). Girls were more likely than boys to present with depressive disorders while boys were more likely to present with ADHD, communication disorders (p values ≤ 0.05, Additional file 3: Table S7 supplemental). A minority of children referred to our outpatient unit (7.9%) were later admitted to the inpatient program. The most common diagnosis associated with admission was disruptive behavior disorders (37.1%) followed by ADHD (17.1%) and depressive disorders (11.4%) (data not shown).

**Table 3:** *Clinical diagnoses of children* seeking psychiatric medical advice according to age groups (n = 886)

Diagnosis	Preschoolers (n = 309, 34.8%)	School age (n = 428, 48.3%)	Adolescents (n = 149, 16.8%)	Total (n = 886)	Statistic	p value
Depressive Disorders	12 (3.9%)^a^	61 (14.3%)^b^	45 (30.2%)^c^	118 (13.3%)	x^2^ = 60.1	*≤ 0.001*
Bipolar disorders	1 (0.3%)	2 (0.5%)	2 (1.3%)	5 (0.6%)	FET	0.4
Anxiety disorders	8 (2.6%)	15 (3.5%)	1 (0.7%)	24 (2.7%)	FET	0.2
Psychotic disorders	0 (0%)	3 (0.7%)	2 (1.3%)	14 (1.6%)	FET	0.06
ADHD	46 (14.9%)^a^	129 (30.1%)^b^	25 (16.8%)^a^	200 (22.6%)	x^2^ = 27.3	*≤ 0.001*
DBD	18 (5.8%)^a^	52 (12.1%)^b^	39 (26.2%)^c^	109 (12.3%)	x^2^ = 38.6	*≤ 0.001*
ASD	45 (14.6%)^a^	7 (1.6%)^b^	0 (0%)^b^	52 (5.9%)	x^2^ = 65.5	*≤ 0.001*
Communication disorders	46 (14.9%)^a^	15 (3.5%)^b^	1 (0.7%)^b^	62 (7%)	x^2^ = 46.7	*≤ 0.001*
OCD spectrum	5 (1.6%)	3 (0.7%)	4 (2.7%)	12 (1.4%)	FET	0.1
Somatic related disorders	1 (0.3%)^a^	2 (0.5%)^a^	4 (2.7%)^c^	7 (0.8%)	FET	*0.04*
Trauma related disorders	0 (0.0%)	4 (0.9%)	2 (1.3%)	6 (0.7%)	FET	0.1
Tics	4 (1.3%)	13 (3.0%)	1 (0.7%)	18 (2%)	FET	0.1
Elimination disorders	21 (6.8%)^a^	55 (12.9%)^b^	12 (8.1%)^a^	88 (9.9%)	x^2^ = 8.1	*0.02*
Intellectual disability	37 (12.0%)	60 (14.0%)	24 (16.1%)	121 (13.7%)	x^2^ = 1.6	0.5
BIF	71 (23%)^a^	151 (35.3%)^b^	32 (21.5%)^a^	254 (28.7%)	x^2^ = 17.8	*≤ 0.001*
Neuropsychiatric disorders	34 (11%)	42 (9.8%)	7 (4.7%)	83 (9.4%)	x^2^ = 4.9	0.08

Differing superscripts denote significant between-group differences calculated using pair-wise Chi square tests (p-values ≤ 0.05)

The exploration of demographic factors associated with certain psychiatric diagnoses yielded the following results. Children with separated parents had the highest risk for depressive disorders (OR = 5.3) followed by those with one parent deceased (OR = 4.4) and then those with divorced parents (OR = 4.2). Sexual abuse (OR = 5.3), physical punishment (OR = 2.4) and having positive family history of any psychiatric illness (OR = 2.6) also increased the risk of a child having a depressive disorder. Children with an ADHD diagnosis were more likely to be physically punished (OR = 1.6) and physically abused (OR 3.4) than other children while a diagnosis of intellectual disability was more likely to be made in families with low income (OR = 2.7), lower educational levels of mothers (OR = 3.3), and in children with history of birth complications (OR = 2.1) and general medical conditions, e.g. Diabetes (OR = 1.8). A history of birth complications was also associated with the diagnosis of ASD (OR = 3.4) while the history of having medical illness was also associated with tics (OR = 3.6). Children with family instability, especially those who lost both parents, were also more likely to develop disruptive behavior disorders (OR = 14.5). Disruptive behavior disorders were also common in higher income families (OR = 14.6) and were associated with high rates of physical punishment (OR = 2.3). Finally, the diagnosis of a psychotic disorder in our sample was more common in middle income families (OR = 4.1) and was associated with family history of psychiatric illness (OR = 3.1) while trauma-related disorders were, as expected, associated with physical (OR = 8.5) and sexual abuse (OR = 21.5) (Table 4).

**Table 4:** Positive (risk) significant odds ratios for diagnostic categories in association with selected demographic/clinical factors

Variable	Depression OR (95% CI)	ADHD OR (95% CI)	ID OR (95% CI)	ASD OR (95% CI)	Tics OR (95% CI)	Disruptive OR (95% CI)	Psychotic OR (95% CI)	Trauma-related OR (95% CI)
Marital status								
Separated	5.3* (1.9–14.6)					–		
Divorced	4.2* (1.7–10.4)					–		
One parent deceased	4.4* (1.9–9.9)					3.3* (1.4–7.9)		
Both parents deceased	–					14.5* (1.3–16.3)		
Family income								
Less than 2500 EGP			2.7* (1.8–4.0)			–	–	
7501 to 10,000 EGP			–			–	4.1* (1.1-15.1)	
15,001 to 20,000 EGP			–			14.6* (2.6 –18.8)		
Mother education								
Illiterate			2.0* (1.2–3.5)					
Primary education			3.3* (1.8–6.0)					
Child abuse								
Physical punishment	2.4* (1.6–3.7)	1.6* (1.1–2.3)				2.3* (1.5 –3.5)		–
Physical abuse	–	3.4* (1.9–6.1)				–		8.5* (1.5 –17.8)
Sexual abuse	5.3* (1.4–19.9)	–				–		21.5* (2.3 –25.4)
History of medical illness (child)			1.8* (1.2–2.8)		3.6* (1.4–9.4)			
Family history of psychiatric illness	2.6* (1.6–4.0)						3.1* (1.0–9.3)	
Birth complications			2.1* (1.4–3.3)	3.4* (1.9–6.1)				

* Significant with p-values ≤ 0.05

## Discussion

To our knowledge, this is largest-to-date study examining Egyptian children seeking psychiatric services. Most of our sample consisted of school aged males coming from well-educated but low to middle income families. When compared to the Egyptian 2018 census data in our catchment area [22], our clinical sample included more males than expected (68.5% vs. 51.2% in population). Our results replicate several previous studies which found that, boys were referred to child psychiatric services twice as frequently as girls [33]. This may reflect the actual higher prevalence of behavioral problems among boys and may also reflect the cultural tendency to give more attention to males than to females, especially in rural areas. On the contrary, other studies [34] documented no gender difference in their clinical samples, while Eapen and her colleagues [17, 18] found significantly higher psychiatric predominance among females in both clinical and community samples in the UAE.

Approximately 75% of our families earned less than 5000 Egyptian pounds (approx. 285 USD) per month, which is close to the average household income in Egypt (3680 EGP, approx. 210 USD, per month) [22]. School aged children were the most represented age category (48.3%) followed by preschoolers (34.8%) and finally adolescents (16.8%). This pattern was similar to that reported by Abdur-Rahim and colleagues [14] in a Saudi Clinical sample, with the exception of our sample having a higher representation of preschoolers. The percentage of preschoolers brought to our clinic was almost equivalent to their percentage among those younger than 18 in the general population (34.8% vs. 36.9% respectively). The under-representation of adolescents in the sample (16.8% vs. 31.6%) may reflect defiance among this age group and their refusal to come to a psychiatric clinic. The relative over-representation of urban families (43.3% vs. 24.8% in population) and the relative under-representation of illiterate parents (10.2% for fathers and 11.2% for mothers versus 27.9% in population) in our sample may reflect the higher tendency of urban and educated families to seek medical services for their children due to higher awareness, higher income, and easier access. Another potential explanation is the controversial finding of lower rates of emotional and behavioral problems in rural children compared to urban children reported and refuted by several international studies that have not always been in agreement [7, 12, 14, 35].

The rates of physical punishment, which is relatively common and accepted in Egyptian culture, reported in the current study (19.8%) is comparable to that previously reported in Yemeni Children [36]. In contrary, our low rates of actual physical and sexual abuse do replicate the results reported by other studies in the Arab world [13] but is different from other reports from non-Arab developing countries. For example, an Indian study [37, 38] found that about 70% of their study subjects faced at least one form of maltreatment. These differences might be explained by dependence of youth self-report in the Indian study and by the tendency of Arab conservative families to under report any form of abuse in their children. Interestingly, sexual abuse was reported almost equally in both sexes, a finding that does not go with the common belief that girls might be more subjected to sexual abuse, as reported by other studies [13, 39]. Conservative communities, especially in rural Egypt, might be more protective to girls and more restrictive when it comes to social interactions leading to less exposure of girls to sexual abuse.

The current study reported higher rates of behavioral disorders (ADHD 22.6% and DBD 12.3%) than emotional disorders (depression 13.3% and anxiety 2.7%) in referred youth. These results are in concordance with the previously reported higher incidence of conduct problems compared to emotional problems (6.5% vs 2%) in large community sample of Egyptian school aged children [19]. The same pattern was reported in other clinical samples from Saudi Arabia [15] and South Africa [13]. As regards the need for admission, cases fulfilling the criteria for admission were mainly youth with sever externalizing behavioral problems. These results replicate previous results [40] which reported that almost one-third of children admitted to inpatient child psychiatric services suffered from disruptive behavior disorders. However, it is to be noted that our inpatient unit lacks the facilities required to admit children with severe neurodevelopmental disorder (e.g. ASD) and that youth with substance abuse were admitted in a separate unit.

As expected from the literature [41, 42], autism spectrum and other communication disorders showed a strong tendency to present in the preschooler age category while ADHD was highly represented in school-age children, and conduct problems were most common in adolescents. Again, as shown in previous studies [7, 43, 44], depressive disorders showed a tendency in the current study to present more frequently in female adolescents. Similar rates of emotional/internalizing disorders in males and females were reported in other Chinese [7] and Yemeni [35] samples but are most probably explained by the predominance of pre-pubertal children in their samples.

In regard to other diagnostic categories, elimination disorders affected 9.9% of the children who attended our mental health care clinic (9.2% male and 11.5 female). This result was in line with that reported by previous studies [13, 45]. The prevalence rate of somatic disorders in our study was 0.8%, close to the previously reported rate of the conversion disorder among children 1–2% [46]. The ratio of obsessional disorder in our finding was 1.4% which is similar to other clinical studies showing that obsessional disorders occurring in 0.2–1.2% of the clinical population of children and adolescents [47].

Our sample did not include any cases with a primary diagnosis of an eating disorder. In general, eating disorders was classically considered less prevalent in non-western developing countries as compared to western developed countries [48, 49], a finding that was attributed to the cultural differences in the perceptions of thinness versus plumpness as symbols of beauty. Although the effect of globalization and social media may increase in the vulnerability to eating disorders in the younger generations [49], a recent study from our area in northern Egypt reported a high prevalence of eating disorders in adult females attending weight management centers [50]. Considering the absence of cases with eating disorder in our sample this finding may reflect a tendency by the public to view eating disorder as nutritional problem rather than a psychiatric one. Other possibilities include the higher prevalence of males in our sample (who have lower prevalence of eating disorders [51, 52]) and the relatively young average age within our sample.

It was reported that the probability of a child suffering from DBD was higher if the child had a deceased mother or separated parents [13]. These results are in concordance with our results that confirmed the correlation between DBD and the loss of one or both parent, marital status of parents, family income as well as physical punishment. Xiaoli et al. [7] noted that externalizing disorders were associated with divorced parents, low SES, and learning disabilities. These results were replicated in numerous studies in developed and developing countries [2, 35, 53–59].

Our study showed that childhood depression was also linked to the marital status of the parents, child abuse as well as the family history of psychiatric illness. Multiple studies showed that a child was prone to mood disorders if they had a deceased parent and/or low family income [13, 60]. The parental depression is highly associated with child psychiatric disorders both due to cross heritability between depression and other psychiatric disorders and because depressed parents have decreased abilities to meet their children needs appropriately [60]. Instability in the family structure after loss of a parent (by death or divorce) and the traumatic consequences after this loss increases the odds of depression in children [61]. These finding highlights the significant effect of family integrity and stability in protecting the offspring from both internalizing and externalizing disorders.

The pattern of associations between physical punishment, physical abuse and sexual abuse with psychiatric disorders in children in our study was very telling. Physical punishment, unfortunately still seen as an acceptable way of discipline especially in rural areas, was significantly associated both with depressive and disruptive disorders in children and adolescents. This replicates several findings from different parts of the world, including Egypt [62–64]. This finding calls for serious effort to build public awareness and legislative action to prevent corporal violence against youth in Egypt and other Arab and Muslim countries. The association between ADHD and both physical punishment and abuse was also expected. It was previously documented that parents of children with ADHD, especially in less developed countries, might use more serious types of physical punishment as compared to those with children without ADHD [65, 66]. The compelling literature about the association between physical and sexual abuse and trauma-related disorders in youth [67] supports findings of our study.

Low family income and low maternal educational level were both associated with intellectual disabilities in children, a finding that replicates previous studies which reported a strong relationship between maternal educational level and ID not associated with major neurological disorders [68]. The lack of similar effect of paternal education on intellectual abilities of the child might be attributed to the higher involvement of mothers in taking care of the children in our community, especially with considering the fact that many fathers travel for work in distant areas inside or outside Egypt and so do not have the chance to spend long times with their children [22]. The association between birth complications, child history of medical illness and neurodevelopmental disorder (i.e. ID and ASD) is not surprising as it is in line with the well-documented relationship between these variables [69, 70].

Before concluding, it is important to highlight the limitations of this study. First, the findings based on a clinical sample from a tertiary hospital, like ours, might reflect community trends in seeking psychiatric help but might not be generalizable to the whole population. Second, most of our patients came from low income families and so our findings might be hard to generalize over the entire population. This could be explained by the low cost of our government-supported service and the tendency of higher income families to seek medical advice in the private health facilities. Third, the diagnoses were made using mainly parent reports and youth self-reports in school-aged children and adolescents but did not include other rater’s reports, such as teachers. Finally, various comorbidities between disorders were not calculated as the study was based only on the primary diagnosis of each case.

## Conclusions

Investigating the demographic and clinical characteristics of children seeking psychiatric care is crucial for planning better future health care services for this age group which forms a sizable percentage of Egypt’s population. The paucity of data about children referred to psychiatric services, and psychiatric services themselves, in developing countries adds to the value of this work. Early assessment and intervention for mental disorders in young populations may decrease the long-term psychological and social burden, particularly delinquency, crime, and substance abuse and improve children’s future functioning.

## Supplementary information

**Additional file 1: Table S1.** Demographic characteristics of children seeking psychiatric medical advice according to gender (n = 886).

**Additional file 2: Table S2.** General clinical characteristics of children seeking psychiatric medical advice according to gender (n = 886).

**Additional file 3: Table S3.** Clinical diagnoses of children seeking psychiatric medical advice according to gender (n = 886).

**Additional file 4.** The dataset created and analyzed during the current study.

## Data Availability

The dataset created and analyzed during the current study will be uploaded with manuscript in excel file format (Additional file 4) and will be available from the corresponding author on reasonable request.

## Abbreviations

ADHD: attention deficit-hyperactivity disorder
ASD: autism spectrum disorder
BIF: borderline intellectual functioning
DBD: disruptive behavior disorders
DSM-5: diagnostic and statistical manual of mental disorders-version 5
ID: intellectual disability
IQ: intelligence quotient
MINI-Kid: MINI International Neuropsychiatric Interview for Children and Adolescents for parents and with children and adolescents
OCD: obsessive compulsive disorder
OR: odds ratio
US: United States
USD: United Stated Dollar

## References

[1] Mary Ellen O’Connell TB, Kenneth EW (2009). Preventing mental, emotional, and behavioral disorders among young people: progress and possibilities.

[2] Costello EJ, Mustillo S, Erkanli A, Keeler G, Angold A (2003). Prevalence and development of psychiatric disorders in childhood and adolescence. Arch Gen Psychiatry.

[3] Jaffee SR, Harrington H, Cohen P, Moffitt TE (2005). Cumulative prevalence of psychiatric disorder in youths. J Am Acad Child Adolesc Psychiatry.

[4] Kessler RC, Berglund P, Demler O, Jin R, Merikangas KR, Walters EE (2005). Lifetime prevalence and age-of-onset distributions of DSM-IV disorders in the National Comorbidity Survey Replication. Arch Gen Psychiatry.

[5] Gregory AM, Caspi A, Moffitt TE, Koenen K, Eley TC, Poulton R (2007). Juvenile mental health histories of adults with anxiety disorders. Am J Psychiatry.

[6] Institute of Medicine (2006). Committee on crossing the quality chasm: adaptation to mental health and addictive disorders. Improving the quality of health care for mental and substance-use conditions: quality chasm series.

[7] Xiaoli Y, Chao J, Wen P, Wenming X, Fang L, Ning L (2014). Prevalence of psychiatric disorders among children and adolescents in northeast China. PLoS ONE.

[8] Patel V, Flisher AJ, Hetrick S, McGorry P (2007). Mental health of young people: a global public-health challenge. Lancet.

[9] Zwirs BW, Burger H, Schulpen TW, Wiznitzer M, Fedder H, Buitelaar JK (2007). Prevalence of psychiatric disorders among children of different ethnic origin. J Abnorm Child Psychol.

[10] Thabet AA, Stretch D, Vostanis P (2000). Child mental health problems in Arab children: application of the strengths and difficulties questionnaire. Int J Soc Psychiatry.

[11] Costello EJ, Egger H, Angold A (2005). 10-year research update review: the epidemiology of child and adolescent psychiatric disorders: I. Methods and public health burden. J Am Acad Child Adolesc Psychiatry..

[12] Mullick MS, Goodman R (2005). The prevalence of psychiatric disorders among 5–10 year olds in rural, urban and slum areas in Bangladesh: an exploratory study. Soc Psychiatry Psychiatr Epidemiol.

[13] Raman N, van Rensburg AB (2013). Clinical and psycho-social profile of child and adolescent mental health care users and services at an urban child mental health clinic in South Africa. Afr J Psychiatry Johannesbg..

[14] Abdur-Rahim FE, Al-Hamd AR, Chaleby K, Al-Subaie A (1995). A survey of a child psychiatry clinic in a teaching hospital in Saudi Arabia—clinical profile and cross-cultural comparison. Saudi Med J..

[15] Al-Modayfer OAY (2015). A pilot study on the prevalence of psychiatric disorders among Saudi children and adolescents: a sample from a selected community in Riyadh City. Arab Psychiatry.

[16] Eapen V, Al-Gazali L, Bin-Othman S, Abou-Saleh M (1998). Mental health problems among schoolchildren in United Arab Emirates: prevalence and risk factors. J Am Acad Child Adolesc Psychiatry..

[17] Eapen V, Al-Sabosy M, Saeed M, Sabri S (2004). Child psychiatric disorders in a primary care Arab population. Int J Psychiatry Med.

[18] Eapen V, Jakka ME, Abou-Saleh MT (2003). Children with psychiatric disorders: the A1 Ain Community Psychiatric Survey. Can J Psychiatry.

[19] Abd-Elhamid A, Howe A, Reading R (2009). Prevalence of emotional and behavioural problems among 6–12 year old children in Egypt. Soc Psychiatry Psychiatr Epidemiol.

[20] Ramy H, El Sheikh M, Sultan M, Bassim R, Eid M, Ali R (2018). Risk factors influencing severity of attention deficit hyperactivity disorder in a sample of preparatory school students in Cairo. Clin Child Psychol Psychiatry..

[21] Meltzer H, Gatward R, Goodman R, Ford T (2003). Mental health of children and adolescents in Great Britain. Int Rev Psychiatry..

[22] (CAPMAS) ECAfPMAS. Egypt in Numbers Cairo. 2018. http://www.capmas.gov.eg.

[23] Hussein H, Shaker N, El-Sheikh M, Ramy HA (2012). Pathways to child mental health services among patients in an urban clinical setting in Egypt. Psychiatr Serv.

[24] ElMakzoum H (2015). Understanding physical punishment as a method of disciplining children in Libya: the perspectives of parents, children and professionals.

[25] Dwairy M, Menshar KE (2006). Parenting style, individuation, and mental health of Egyptian adolescents. J Adolesc..

[26] Egyptian Child Act. Original Act 1996. 2008.

[27] Sheehan DLY, Sheehan K, Janavs J, Weiller E, Keskiner A (1997). The validity of the Mini International Neuropsychiatric Interview (MINI) according to the SCID-P and its reliability. Eur Psychiatry..

[28] Ghanem S (1998). Mini Kid schedule semi-structured interview.

[29] Ibrahim MBZ, Hamed A. Comparison of Mini International Neuropsychiatric Interview for children (MINI-KID) with the schedules for affective disorders and schizophrenia for schoolaged children, present and lifetime version (KSADS-PL). In: Egyptian sample presenting with childhood disorders. Cairo: Ain Shams University; 2002.

[30] Association AP (2013). Diagnostic and statistical manual of mental disorders.

[31] Thorndike RLHE, Sattler M (1986). Stanford-binet intelligence scale.

[32] The Melika L, Scale Stanford Binet Intelligence (1998). Arabic Examiner’s Handbook.

[33] Qureshi E-R (1988). A psychiatric clinic in a primary care setting: evaluating the experience. Saudi Med J..

[34] El-Rufaie OEAG (1993). Minor psychiatric morbidity in primary health care: prevalence, nature and severity. Int J Soc Psychiatry.

[35] Alyahri A, Goodman R (2008). The prevalence of DSM-IV psychiatric disorders among 7–10 year old Yemeni schoolchildren. Soc Psychiatry Psychiatr Epidemiol.

[36] Alyahri A, Goodman R (2008). Harsh corporal punishment of Yemeni children: occurrence, type and associations. Child Abuse Negl.

[37] Daral S, Khokhar A, Pradhan S (2016). Prevalence and determinants of child maltreatment among school-going adolescent girls in a semi-urban area of Delhi, India. J Trop Pediatr..

[38] Daral S, Khokhar A, Pradhan SK (2016). Barriers to disclosure of child maltreatment among school-going adolescent girls of a semi-urban area of Delhi, India. Int J Adolesc Med Health..

[39] Joseph CCJ, Eckenrode J, Powers JL (1993). The epidemiology of child abuse: findings from the Second National Incidence and prevalence study of Child Abuse and Neglect. Am J Public Health.

[40] Moodley SV, Pillay AL (1993). Two years of admissions to Natal’s first inpatient child mental health centre. S Afr Med J.

[41] Kessler RC, Avenevoli S, Costello EJ, Georgiades K, Green JG, Gruber MJ (2012). Prevalence, persistence, and sociodemographic correlates of DSM-IV disorders in the National Comorbidity Survey Replication Adolescent Supplement. Arch Gen Psychiatry.

[42] Mandell DS, Morales KH, Xie M, Lawer LJ, Stahmer AC, Marcus SC (2010). Age of diagnosis among medicaid-enrolled children with autism, 2001–2004. Psychiatr Serv.

[43] Merikangas KR, He JP, Brody D, Fisher PW, Bourdon K, Koretz DS (2010). Prevalence and treatment of mental disorders among US children in the 2001–2004 NHANES. Pediatrics.

[44] Merikangas KR, He JP, Burstein M, Swanson SA, Avenevoli S, Cui L (2010). Lifetime prevalence of mental disorders in U.S. adolescents: results from the National Comorbidity Survey Replication-Adolescent Supplement (NCS-A). J Am Acad Child Adolesc Psychiatry..

[45] Street EBI (1990). The treatment of childhood nocturnal enuresis in the community. Child Care Health Dev.

[46] Gooyer I (1981). Hystencal conversion reaction in childhood. J Child Psychol Psychiatry..

[47] Hollingsworth CETP, Grossman L, Past P (1980). Longtern outcome of obsessive-compulsive disorders in childhood. J Am Acad Child Psychiatry..

[48] Nasser M (1994). Screening for abnormal eating attitudes in a population of Egyptian secondary school girls. Soc Psychiatry Psychiatr Epidemiol.

[49] Makino M, Tsuboi K, Dennerstein L (2004). Prevalence of eating disorders: a comparison of Western and non-Western countries. MedGenMed..

[50] Eladawi N, Helal R, Niazy NA, Abdelsalam S (2018). Prevalence and associated factors of eating disorders in weight management centers in Tanta, Egypt. Chin Med J (Engl).

[51] Hoek HW (2016). Review of the worldwide epidemiology of eating disorders. Curr Opin Psychiatry..

[52] Mitchison D, Mond J (2015). Epidemiology of eating disorders, eating disordered behaviour, and body image disturbance in males: a narrative review. J Eat Disord..

[53] Guan BQ, Luo XR, Deng YL, Wei Z, Ye HS, Yuan XH (2010). Prevalence of psychiatric disorders in primary and middle school students in Hunan Province. Zhongguo Dang Dai Er Ke Za Zhi..

[54] Ford T, Goodman R, Meltzer H (2003). The British Child and Adolescent Mental Health Survey 1999: the prevalence of DSM-IV disorders. J Am Acad Child Adolesc Psychiatry.

[55] Frigerio A, Rucci P, Goodman R, Ammaniti M, Carlet O, Cavolina P (2009). Prevalence and correlates of mental disorders among adolescents in Italy: the PrISMA study. Eur Child Adolesc Psychiatry.

[56] Heiervang E, Stormark KM, Lundervold AJ, Heimann M, Goodman R, Posserud MB (2007). Psychiatric disorders in Norwegian 8- to 10-year-olds: an epidemiological survey of prevalence, risk factors, and service use. J Am Acad Child Adolesc Psychiatry.

[57] Goodman R, Slobodskaya H, Knyazev G (2005). Russian child mental health—a cross-sectional study of prevalence and risk factors. Eur Child Adolesc Psychiatry.

[58] Canino G, Shrout PE, Rubio-Stipec M, Bird HR, Bravo M, Ramirez R (2004). The DSM-IV rates of child and adolescent disorders in Puerto Rico: prevalence, correlates, service use, and the effects of impairment. Arch Gen Psychiatry.

[59] Goodman R, Neves dos Santos D, Robatto Nunes AP, Pereira de Miranda D, Fleitlich-Bilyk B, Almeida Filho N (2005). The Ilha de Mare study: a survey of child mental health problems in a predominantly African–Brazilian rural community. Soc Psychiatry Psychiatr Epidemiol..

[60] Restifo K, Bogels S (2009). Family processes in the development of youth depression: translating the evidence to treatment. Clin Psychol Rev..

[61] Dowdney L (2000). Childhood bereavement following parental death. J Child Psychol Psychiatry.

[62] Csorba J, Rozsa S, Vetro A, Gadoros J, Makra J, Somogyi E (2001). Family- and school-related stresses in depressed Hungarian children. Eur Psychiatry..

[63] Hecker T, Hermenau K, Isele D, Elbert T (2014). Corporal punishment and children’s externalizing problems: a cross-sectional study of Tanzanian primary school aged children. Child Abuse Negl.

[64] Youssef RM, Attia MS, Kamel MI (1998). Children experiencing violence I: parental use of corporal punishment. Child Abuse Negl..

[65] Blachno M, Szamanska U, Kolakowski A, Pisula A (2006). Parental corporal punishment in children with attention-deficit hyperactivity syndrome. Psychiatr Pol.

[66] Shin DW, Stein MA (2008). Maternal depression predicts maternal use of corporal punishment in children with attention-deficit/hyperactivity disorder. Yonsei Med J.

[67] Mitchell R, Brennan K, Curran D, Hanna D, Dyer KF (2017). A meta-analysis of the association between appraisals of trauma and posttraumatic stress in children and adolescents. J Trauma Stress.

[68] Decoufle P, Boyle CA (1995). The relationship between maternal education and mental retardation in 10-year-old children. Ann Epidemiol.

[69] Soleimani F, Zaheri F, Abdi F (2014). Long-term neurodevelopmental outcomes after preterm birth. Iran Red Crescent Med J..

[70] Wang C, Geng H, Liu W, Zhang G (2017). Prenatal, perinatal, and postnatal factors associated with autism: a meta-analysis. Medicine (Baltimore)..

