# Supplementary material for: Demographic and clinical characteristics of children seeking psychiatric services in the Nile Delta region: an observational retrospective study
Source: Int J Ment Health Syst. 2019 Oct 23;13:66. doi: 10.1186/s13033-019-0323-6 (PMC6806528; doi:10.1186/s13033-019-0323-6)
Supplement: Supplementary file 1 — Additional file 1: Table S1. Demographic characteristics of children seeking psychiatric medical advice according to gender (n = 886). [file 13033_2019_323_MOESM1_ESM.docx]

**Table (S1): *Demographic characteristics of children* seeking psychiatric medical advice according to gender (n=886)**

| p value | Statistic | Total (n=886) | Female  (n=279, 31.5%) | Male  (n= 607, 68.5%) | Variable | |
| --- | --- | --- | --- | --- | --- | --- |
| 0.06 | t = 1.9 | 7.5 ± 3.8 | 7.9 ± 4.1 | 7.4 ± 3.7 | Age | |
| 0.5 | x^2^ = 0.5 | 502 (56.7%) | 163 (58.4%) | 339 (55.9%) | Rural | Residence |
|  |  | 383 (43.3%) | 116 (41.6%) | 267 (44.1%) | Urban |  |
| 0.8 | x^2^ = 0.06 | 797 (90%) | 252 (90.3%) | 545 (89.8%) | Family Status  (Living with Both Parents) | |
| 0.2 | x^2^ = 4.2 | 90 (10.2%) | 36 (12.9%) | 54 (8.9 %) | Illiterate | Father Education |
|  |  | 126 (14.2%) | 43 (15.4%) | 83 (13.7%) | < High school |  |
|  |  | 670 (75.6%) | 200 (71.7%) | 470 (77.4%) | ≥ High school |  |
| 0.9 | x^2^ = 0.3 | 99 (11.2%) | 32 (11.5%) | 67 (11 %) | Illiterate | Mother Education |
|  |  | 152 (17.2%) | 45 (16.1%) | 107 (17.6%) | < High school |  |
|  |  | 635 (71.1%) | 202 (72.4%) | 433 (71.3%) | ≥ High school |  |
| 0.7 | x^2^ = 0.2 | 666 (75.2%) | 212 (76%) | 454 (74.8%) | < 5000 EGP / M | Family Income |
|  |  | 220 (24.8%) | 67 (24%) | 153 (25.2%) | ≥ 5000 EGP / M |  |
| 0.5 | FET | 300 (34.1%) | 89 (31.9%) | 211 (35.2%) | Out of school | Child Education |
|  |  | 566 (64.4%) | 187 (67%) | 379 (63.2%) | Regular school |  |
|  |  | 13 (1.5%) | 3 (1.1%) | 10 (1.7%) | Special school |  |
