# Supplementary material for: Demographic and clinical characteristics of children seeking psychiatric services in the Nile Delta region: an observational retrospective study
Source: Int J Ment Health Syst. 2019 Oct 23;13:66. doi: 10.1186/s13033-019-0323-6 (PMC6806528; doi:10.1186/s13033-019-0323-6)
Supplement: Supplementary file 2 — Additional file 2: Table S2. General clinical characteristics of children seeking psychiatric medical advice according to gender (n = 886). [file 13033_2019_323_MOESM2_ESM.docx]

**Table (S2): *General Clinical characteristics of children* seeking psychiatric medical advice according to gender (n=886)**

| p value | Statistic | Total (n=886) | Female  (n=279, 31.5%) | Male  (n= 607, 68.5%) | Variable | |
| --- | --- | --- | --- | --- | --- | --- |
| 0.6 | t = 0.5 | 28.4 ± 14.0 | 28.9 ± 13.3 | 28.2 ± 14.4 | Weight | |
| 0.1 | x^2^ = 0.1 | 137 (15.6%) | 51 (18.4%) | 86 (14.4%) | Family History of Psychiatric Illness | |
| 0.4 | x^2^ = 0.8 | 158 (18.1%) | 45 (16.3%) | 113 (18.9%) | History of Birth Complications | |
| 0.9 | x^2^ = 0.03 | 394 (45.1%) | 126 (45.5%) | 268 (44.9%) | History of Delayed Milestones | |
| 0.2 | x^2^ = 1.9 | 17 (1.9%) | 8 (2.9%) | 9 (1.5%) | Birth Order (5^th^ or more) | |
| 0.6 | FET | 137 (19.8%) | 48 (17.4%) | 125 (20.9 %) | Physical Punishment | History of Abuse |
|  |  | 50 (5.7%) | 17 (6.2%) | 33 (5.5%) | Physical Abuse |  |
|  |  | 9 (1%) | 2 (0.7%) | 7 (1.2%) | Sexual Abuse |  |
| 0.1 | x^2^ = 2.6 | 70 (7.9%) | 16 (5.7%) | 54 (8.9%) | Need for admission | |
