# Supplementary material for: Demographic and clinical characteristics of children seeking psychiatric services in the Nile Delta region: an observational retrospective study
Source: Int J Ment Health Syst. 2019 Oct 23;13:66. doi: 10.1186/s13033-019-0323-6 (PMC6806528; doi:10.1186/s13033-019-0323-6)
Supplement: Supplementary file 3 — Additional file 3: Table S3. Clinical diagnoses of children seeking psychiatric medical advice according to gender (n = 886). [file 13033_2019_323_MOESM3_ESM.docx]

**Table (S3): *Clinical diagnoses of children* seeking psychiatric medical advice according to gender (n=886)**

| p value | Statistic | Total (n=886) | Female  (n=279, 31.5%) | Male  (n= 607, 68.5%) | Diagnosis |
| --- | --- | --- | --- | --- | --- |
| **≤ 0.001** | x^2^ = 14.4 | 118 (13.3%) | 55 (19.7%) | 63 (10.4%) | Depressive Disorders |
| 0.6 | FET | 5 (0.6%) | 2 (7%) | 3 (0.5%) | Bipolar Disorders |
| 0.5 | x^2^ = 0.5 | 24 (2.7%) | 6 (2.2%) | 18 (3%) | Anxiety Disorders |
| 0.3 | FET | 14 (1.6%) | 2 (0.7%) | 12 (2%) | Psychotic Disorders |
| **0.002** | x^2^ = 9.7 | 200 (22.6%) | 45 (16.1%) | 155 (25.5%) | ADHD |
| 0.1 | x^2^ = 2.6 | 109 (12.3%) | 27 (9.7%) | 82 (13.5%) | DBD |
| 0.9 | x^2^ = 0.01 | 52 (5.9%) | 16 (5.7%) | 36 (5.9%) | ASD |
| **0.003** | x^2^ = 8.9 | 62 (7%) | 9 (3.2%) | 53 (8.7%) | Communication Disorders |
| 0.6 | FET | 12 (1.4%) | 7 (2.5%) | 5 (0.8%) | OCD spectrum |
| 0.2 | FET | 7 (0.8%) | 4 (1.4%) | 3 (0.5%) | Somatic Related Disorders |
| 1 | FET | 6 (0.7%) | 2 (0.7%) | 4 (0.7%) | Trauma Related Disorders |
| **0.02** | x^2^ = 4.9 | 18 (2%) | 10 (3.6%) | 8 (1.3%) | Tics |
| 0.3 | x^2^ = 1.1 | 88 (9.9%) | 32 (11.5%) | 56 (9.2%) | Elimination Disorders |
| 0.2 | x^2^ = 2.1 | 121 (13.7%) | 45 (16.1%) | 76 (12.5%) | Intellectual Disability |
| 0.8 | x^2^ = 0.1 | 254 (28.7%) | 78 (28%) | 176 (29%) | BIF |
| 0.5 | FET | 9 (1%) | 2 (0.7%) | 7 (1.2%) | Global Developmental Delay |
| 0.05 | x^2^ = 3.8 | 83 (9.4%) | 34 (12.2%) | 49 (8.1%) | Neuropsychiatric Disorders |
